# Effect of delayed remote ischemic preconditioning on major cardiovascular and cerebrovascular events in major vascular surgery: the protocol of DRIPC randomized controlled trial

**DOI:** 10.3389/fcvm.2026.1952010

**Published:** 2026-10-08

**Authors:** Linyao Zhu, Min Li, Jichun Zhao, Bin Huang, Ding Yuan, Chengxin Weng, Tiehao Wang, Huawei Zhang, Jiarong Wang

**Affiliations:** 1 Division of Vascular Surgery, Department of General Surgery, West China Hospital, Sichuan University, Chengdu, China; 2 West China School of Medicine, Sichuan University, Chengdu, China; 3 Department of Radiology, Huaxi MR Research Center (HMRRC), Functional and Molecular Imaging Key Laboratory of Sichuan Province, West China Hospital, Sichuan University, Chengdu, China

**Keywords:** AKI (acute kidney injury), MACCE (major adverse cardiac and cerebrovascular events), RCT - randomized controlled trial, vascular surgery, delayed remote ischemic preconditioning (DRIPC)

## Abstract

**Introduction:**

Although endovascular therapy has become the mainstream approach for major vascular surgery, patients undergoing such procedures are still classified as high-risk according to clinical guidelines. Remote ischemic preconditioning (RIPC) is a promising perioperative intervention strategy that has demonstrated acceptable safety and efficacy. Existing research indicates that delayed remote ischemic preconditioning (DRIPC) offers superior protective effects compared to preconditioning performed within 24 h before surgery. However, there is currently a lack of Level I evidence regarding the efficacy and safety of DRIPC in the perioperative period for vascular surgery patients. This study aims to provide high-level evidence on the effectiveness and safety of this intervention in a clinical setting.

**Methods and analysis:**

The trial is a single-center, prospective, double-blind, randomized controlled clinical trial. A total of 1,054 patients will be recruited. The primary endpoints of the study include major adverse cardiac and cerebrovascular events (MACCE) within 30 days postoperatively.

**Ethics and dissemination:**

This study has been approved by the Ethics Committee of West China Hospital of Sichuan University (approval number:2025-1846). The results will be submitted to a major clinical journal for peer review and publication.

**Clinical Trial registration:**

identifier ChiCTR2600116987

## Strengths and limitations of this study

1. High patient engagement, well-structured follow-up periods, and a low rate of loss to follow-up.
2. This trial one of the earlist randomized controlled trials specifically designed to evaluate the clinical efficacy of DRIPC in patients undergoing major vascular surgery.
3. Conducted in China, this trial may introduce potential bias due to regional population characteristics, healthcare practice patterns, and disease spectra, which could affect the interpretability of the findings and their applicability to other healthcare systems.

## Introduction

Major vascular disease is broadly categorized into two principal categories: aortic pathologies and peripheral artery disease. Predominantly driven by systemic atherosclerosis, these conditions pose substantial threats to limb viability, overall survival, and global healthcare systems. Consequently, timely and appropriate intervention is of paramount importance. Although endovascular therapy has become the primary treatment modality in contemporary vascular surgery due to their minimally invasive nature and expedited recovery profiles, the 2002 and 2024 ACC/AHA guidelines continue to classify these procedures as high-risk (1, 2). This classification underscores the formidable burden of perioperative adverse cardiovascular and systemic complications in this patient population.

Therefore, in addition to conducting thorough preoperative evaluations and maintaining meticulous operative technique, effective adjunctive organ-protective strategies are urgently needed to optimize clinical outcomes (3). In this context, remote ischemic preconditioning (RIPC) has emerged as an attractive, non-pharmacological paradigm for systemic organoprotection.

RIPC is a feasible, non-invasive clinical intervention. This intervention involves inducing transient cycles of ischemia and reperfusion in a limb, typically the upper arm, thereby triggering the release of circulating humoral factors. These mediators subsequently initiate complex intracellular signaling cascades, conferring remote resistance against ischemia-reperfusion injury and preserving systemic homeostasis (3, 4).

Translational interest in RIPC has grown rapidly, reflected by nearly 400 publications over the past five years, including 34 randomized controlled trials (RCTs) specifically in non-cardiac surgical settings (5). The efficacy of RIPC has been robustly established in preclinical animal models of organ protection (6). Furthermore, a substantial body of clinical evidence, encompassing numerous RCTs and associated meta-analyses, indicates that RIPC may confer tangible improvements in patient-centered outcomes (7). Accordingly, consensus reviews have recognized RIPC as a viable perioperative strategy to attenuate perioperative myocardial injury (8).

Notably, the paradigm of the “second window of protection” differentiates delayed RIPC (DRIPC), applied 24 to 72 h preoperatively, from the more extensively investigated acute RIPC (9). It has been posited that the delayed variant confers more robust cardio-protection, extending its benefits against both infarction and iatrogenic cardiac injury (9, 10). Despite encouraging safety and efficacy profiles across various non-cardiac cohorts, a critical evidence gap remains regarding the impact of DRIPC on perioperative outcomes specifically in patients undergoing major vascular surgery.

Therefore, this single-center, prospective, randomized controlled trial was designed to investigate the effect of DRIPC on the incidence of perioperative major adverse cardiac and cerebrovascular events, acute kidney injury, and all-cause mortality in patients undergoing major vascular surgery. The findings are intended to provide high-level clinical evidence to inform perioperative organoprotective strategies in modern vascular surgery.

## Method and analysis

### Study design

This single-center, prospective, double-blind, randomized controlled trial will be conducted at West China Hospital, Sichuan University, between October1, 2026, and October 1, 2027. A total of 1,054 participants will be randomly assigned in a 1:1 ratio to either the intervention group or the control group. All investigators and research assistants will undergo standardized training to ensure procedural consistency. Data for all enrolled patients will be recorded using standardized case report forms and a postoperative follow-up questionnaire. The primary outcome assessment will be patient-centered, and the follow-up modality will be determined according to individual patient preference.

This trial will adhere to the Standard Protocol Items: Recommendations for Interventional Trials (SPIRIT) guidelines. The SPIRIT flow diagram and checklist are presented in Figure 1 and Additional file 1, respectively. The participant flow diagram is detailed in Figure 1, and the schedule of enrollment, interventions, and assessments is presented in Table 1.

**Figure 1:**
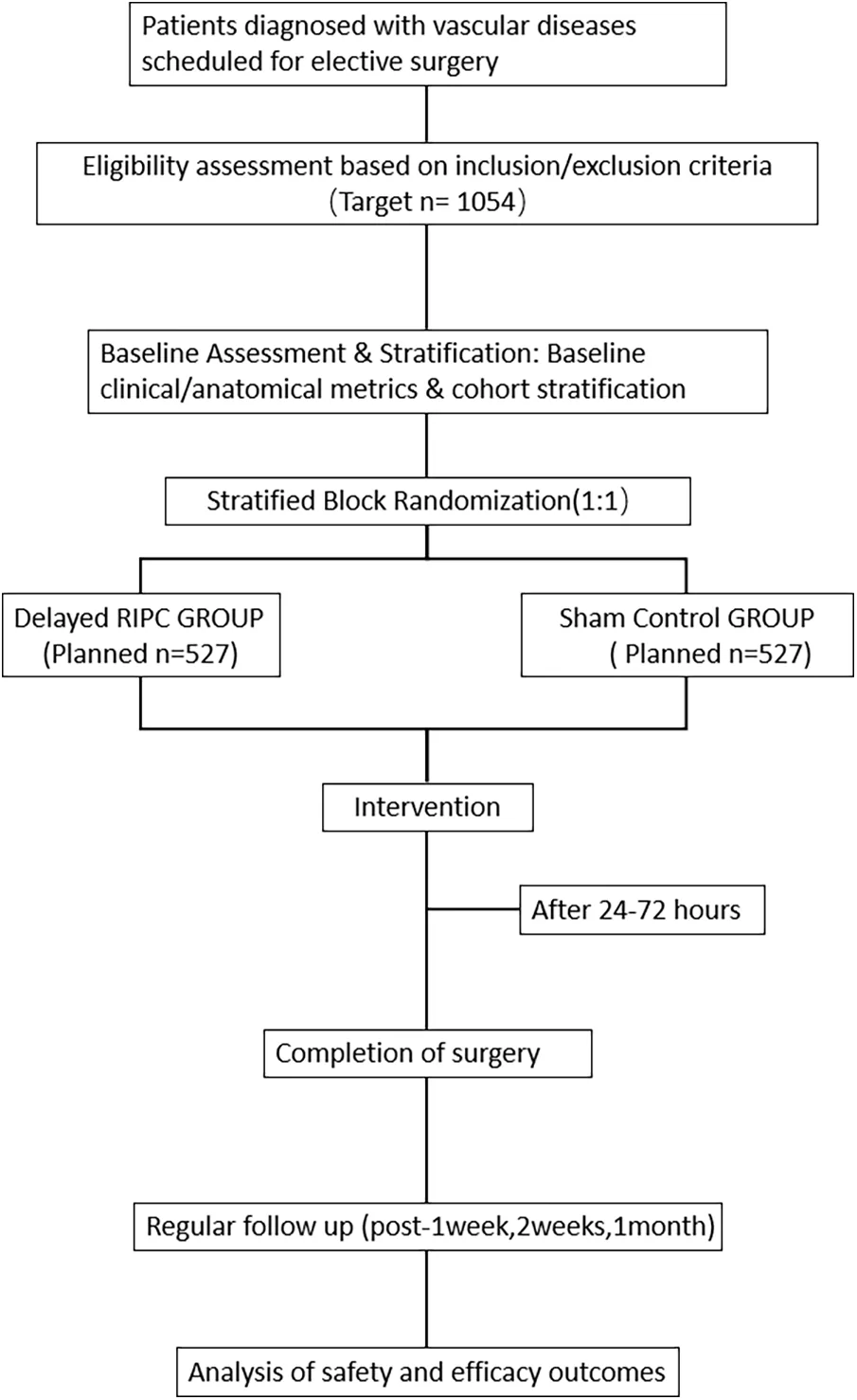
The procedure of screening, randomization and follow-up of patients in the trial.

**Table 1:** The schedule of enrollment, interventions and assessments.

	Study period						
	Enrollment	Allocation	Post allocation		Close-out		
Time point		0	prior to surgery	perioperative period(48 h)	t1 (7 days))	t2 (15-days)	t3 (30 days)
Enrollment:							
Eligibility screen	√						
Informed consent	√						
Demographic characteristics and medical history	√						
Allocation		√					
Intervention:							
DRIPC			√				
Standard control			√				
Assessments:							
Major adverse cardiac and cerebrovascular events				√	√	√	√
Acute Kidney Injury				√	√	√	√
30-day overall complication rate							√
Time to return to normal activities				√	√	√	√
Postoperative length of stay				√			
ICU length of stay				√			
Quality of life				√	√	√	√

### Recruitment

On obtaining informed consent, participants will be recruited from outpatient department, emergency department and through posters or online advertisements to achieve the required sample size.

### Inclusion criteria

1. Aged between 18 and 90 years.
2. Scheduled to undergo moderate- to high-risk vascular surgery (defined by the 2002 ACC/AHA guidelines, including open or endovascular procedures involving the aorta, carotid, visceral, or lower extremity arteries, or retroperitoneal tumor resections with major vascular reconstruction (1).
3. Ability to understand the study procedures and provide written informed consent.
4. No restrictions regarding pre-existing comorbidities such as hypertension, diabetes mellitus, or coronary artery disease.

### Exclusion criteria

1. Requiring emergency surgical intervention.
2. Presence of upper limb fracture or significant upper extremity vascular disease.
3. Acute myocardial infarction (MI) within the preceding 1 month or unstable angina.

1) Acute MI is defined by the presence of one or more of the following: new or presumed new significant ST-T wave changes or new left bundle branch block on ECG; appearance of pathological Q waves; imaging evidence of new loss of viable myocardium or new regional wall motion abnormality; or identification of an intracoronary thrombus by angiography or autopsy (11).
2) Unstable Angina is defined clinically as rest or crescendo angina in the absence of myocardial necrosis, supported by objective evidence of myocardial ischemia (e.g., transient ST-segment or T-wave changes on ECG) and the absence of elevated cardiac biomarkers (e.g., troponin) (12, 13).

4. Acute stroke within the preceding 30 days. (Stroke is defined as an acute episode of focal cerebral, retinal, or spinal cord dysfunction lasting >24 h or of any duration with corresponding infarction or hemorrhage on neuroimaging (CT or MRI) or autopsy. This includes patients presenting with clinical symptoms such as unilateral weakness/numbness, visual loss, diplopia, speech disturbance, ataxia, or non-positional vertigo (14).)
5. Pre-existing end-stage renal disease (ESRD), defined as a baseline estimated glomerular filtration rate (eGFR) < 15 mL/min/1.73 m^2^ calculated using the CKD-EPI equation, or receipt of maintenance renal replacement therapy (hemodialysis or peritoneal dialysis) prior to surgery.

To maintain the representativeness of the real-world vascular surgery population, pre-existing chronic renal insufficiency was not set as an exclusion criterion. Instead, in accordance with KDIGO criteria, postoperative acute kidney injury (AKI) will be determined based on dynamic changes in serum creatinine and eGFR relative to each patient's preoperative baseline values. Baseline renal function will be recorded and accounted for as a stratification factor during randomization and a covariate in adjusted analyses.

### Criteria for dropout

1. Poor compliance by subjects, such as being lost to follow-up after surgery;
2. Participants withdrawal from the trial for any reason;

### Study termination criteria

1. The subject presents a safety risk requiring immediate termination.
2. High voluntary withdrawal and poor compliance affecting the study;
3. High number of patients no longer followed up or receiving treatment.

### Randomization and allocation

The study population will comprise three independent cohorts: the aortic cohort, the peripheral arterial cohort, and the retroperitoneal tumor cohort. To prevent baseline imbalances across cohorts and key prognostic factors in this prospective RCT, randomization will be executed using stratified block randomization (with a block size of 4 or 6) generated by R software (version 4.5.1), stratified by cohort type (aortic, peripheral arterial, and retroperitoneal tumor cohorts). Participants will be randomly assigned in a 1:1 ratio to either the intervention group or the control group according to a computer-generated random number sequence. The random sequence will be generated and allocation codes will be managed by independent personnel who are not involved in participant recruitment or outcome assessment, thereby ensuring objectivity and independence of the randomization process. The allocation sequence will be concealed using sealed, opaque, sequentially numbered envelopes. After baseline assessments are completed, an unblinded assistant will sequentially inform the operating nurses of the randomization outcome prior to surgery. Both the patients and the surgeons will remain blinded to group allocation. Unblinding will be permitted only after trial completion or if a participant experiences a serious adverse event requiring emergency treatment. To ensure analytical independence and objectivity, an independent statistician who remains blinded to group allocation will perform the statistical analyses. The specific unblinding procedures, including the timing, sequence, and process for verifying participant identity, will be established and approved by the Ethics Committee.

### Study intervention

Prior to the intervention, written informed consent will be obtained from all participants or their legally authorized representatives. All enrolled patients will receive standardized perioperative care, which will include protocols for blood pressure management and analgesic administration. Furthermore, comprehensive baseline assessments, including computed tomography angiography (CTA) of the head and neck and transthoracic echocardiography, will be performed to evaluate underlying comorbidities and cerebrovascular status in this cohort. Based on these findings, the choice between general and regional anesthesia will be determined by the attending anesthesiologists.

All interventions will be administered 24 to 72 h prior to surgery. Patients in the DRIPC group will undergo three consecutive cycles of 5-minute ischemia followed by 5-minute reperfusion using a pneumatic cuff placed on a single upper arm. The cuff will be inflated to 200 mmHg, or 50 mmHg above the baseline systolic blood pressure (SBP) for patients with SBP ≤150 mmHg. For patients with baseline SBP >180 mmHg, blood pressure will be pharmacologically controlled below 180 mmHg prior to protocol initiation. Patients in the sham-control group will undergo an identical three-cycle maneuver with the cuff inflated to an ineffective pressure of 50 mmHg, which generates sensory perception without inducing distal ischemia. Furthermore, to formally evaluate blinding success, a structured blinding questionnaire will be administered to all participants at 24 h after the procedure. Participants will be asked: “Which intervention do you believe you received?” with the following response options: (1) Active RIPC, (2) Sham procedure, or (3) Do not know/Unsure. Blinding success and the direction of potential unblinding will be quantitatively assessed using Bang's Blinding Index and James’ Blinding Index. Surgeons, ward physicians, outcome adjudicators, and data analysts will remain strictly blinded throughout the trial.

Standardized perioperative pathways, including hemodynamic goals, analgesia protocols, and comprehensive baseline evaluations (e.g., computed tomographic angiography and transthoracic echocardiography), will be uniformly enforced across all cohorts. Surgical strategies will be finalized via multidisciplinary team (MDT) consensus and supervised by the Institutional Surgical Quality Assurance Committee.

### Intervention modifications and adherence

During the intervention, the operating nurse will continuously monitor limb perfusion and subject tolerance. The procedure will be terminated immediately if serious adverse events occur or if the participant requests discontinuation because of intolerance. In accordance with the intention-to-treat (ITT) principle, participants who discontinue the intervention prematurely will not be excluded from the trial. They will remain in the study, continue to receive standard perioperative care, and complete all scheduled clinical and safety follow-up assessments. Their outcomes will be retained in the primary ITT analysis according to their randomized allocation, while being documented as protocol non-adherence and evaluated separately in the secondary per-protocol (PP) sensitivity analysis. Non-substantial protocol modifications will be documented by the research team and will not require notification of the monitoring committee or regulatory authorities. To enhance adherence to the intervention and follow-up protocols, the principal investigator will maintain regular contact with clinical investigators via telephone or WeChat to provide reminders and support. Furthermore, a dedicated follow-up coordinator will facilitate communication with the surgical team as needed. All enrolled participants will be provided with free outpatient clinic access to promote retention and facilitate complete data collection.

### Follow-up

Following the initial intervention, participants will have access to both outpatient clinic visits and remote follow-up modalities (via telephone or WeChat). Blood samples for the assessment of myocardial enzymes, B-type natriuretic peptide (BNP), and renal function will be collected on postoperative days 2, 7, 15, and 30. Serum high-sensitivity cardiac troponin T (hs-cTnT) will be measured using the Roche Elecsys hs-cTnT assay has a 99th percentile upper reference limit of 14 ng/L. Blood samples will be collected from all participants at the following prespecified time points: Preoperative: within 24 h before surgery as the baseline measurement; Postoperative: at 48 h (± 4 h), and 7 days (± 4 h); Immediate additional measurement whenever any symptoms or signs of acute ischemia occur. All suspected cardiac events will be centrally reviewed and adjudicated by an independent clinical endpoint committee (CEC) under blinded conditions.

Urine output will be monitored daily during the first 7 postoperative days. Questionnaire-based assessments will be conducted on postoperative day 15. The primary study endpoint will be 30 days postoperatively. During follow-up, arterial duplex ultrasound will serve as the primary non-invasive vascular imaging modality for all participants. Postoperative CTA and transthoracic echocardiography will not be mandatory for all participants; rather, they will be performed selectively according to disease-specific clinical practice guidelines (e.g., routine CTA surveillance after aortic stent-graft repair) or when clinically indicated (e.g., suspected acute regional ischemia, vessel or graft occlusion, endoleak, or new-onset cardiac dysfunction) to support accurate endpoint adjudication. All postoperative medications will be administered according to clinical necessity without protocol-specified restrictions.

### Post-trial care

Based on evidence from previous clinical studies, no underlying harm is anticipated for trial participants undergoing DRIPC. Following the intervention, participants will receive guidance regarding specialized postoperative care protocols and wound dressing management.

### Outcomes of interest

#### Primary outcome

The primary endpoint is the composite incidence of major adverse cardiac and cerebrovascular events (MACCE) occurring within 30 days postoperatively. To maintain clinical rigor and avoid heterogeneity in event severity, MACCE is defined as a composite hard clinical endpoint comprising all-cause mortality, non-fatal myocardial infarction (adjudicated according to the Fourth Universal Definition of Myocardial Infarction (15), heart failure requiring urgent intervention/hospitalization, stroke, and systemic embolism; isolated asymptomatic biomarker elevation is excluded from the primary MACCE composite. Since this outcome is a composite endpoint, each individual component event will be recorded separately and also included in the analyses of other relevant outcome measures.

#### Secondary outcome

1) AKI is defined as meeting any of the following criteria within 7 days postoperatively: an increase in serum creatinine by ≥0.3 mg/dL (≥26.5 μmol/L) within 48 h; an increase in serum creatinine to ≥1.5 times the baseline value; or a sustained urine output of <0.5 mL/kg/h for at least 6 h (16).
2) 30-day overall complication rate

#### Other outcomes

1) Health-related quality of life at 30 days, assessed using the EQ-5D-5L questionnaire.
2) Time to return to normal activities (days)
3) Postoperative length of stay (days)
4) ICU length of stay (days)
5) Procedure-related adverse events: Including skin bruising at the cuff site and limb ischemia.
6) Myocardial infarction,
7) Heart failure
8) A rise in troponin levels to more than twice the upper limit of normal
9) Stroke
10) Systemic embolism
11) All-cause mortality.

### Sample size calculation

Based on data reported by Fresilli et al. (7), the individual incidences of stroke and myocardial infarction in the RIPC group were 1.6% (13/793) and 7.7% (87/1,132), respectively, compared with 3.8% (32/849) and 10.0% (113/1,128) in the control group. Because our target population consists of high-risk vascular surgery patients with comparable baseline atherothrombotic and cerebrovascular profiles, the composite 30-day primary endpoint rate was estimated by summing the event rates of stroke and myocardial infarction for each group, yielding an anticipated rate of 9.33% in the RIPC group and 13.79% in the control group. Sample size calculation will be performed using the “Test for Two Proportions” module in PASS software (version 15.0.5). Assuming a two-sided *Z*-test for pooled proportions, a significance level (*α*) of 0.05 and a statistical power of 80% (1−*β* = 0.80) with a 1:1 randomization ratio, a minimum of 421 participants per group will be required. To ensure adequate power while accounting for potential protocol non-adherence, withdrawal, and loss to follow-up, conservatively anticipated at 20% because of the advanced age, multimorbidity, and multiple postoperative follow-up time points in this vascular surgery cohort, the final enrollment target was set at 527 participants per group, yielding a total required sample size of 1,054 participants. Recruitment will be conducted through posters, online advertisements, and direct outreach in outpatient clinics and emergency departments.

### Data collection and management

Demographic characteristics, including age, sex, body mass index, comorbidities, and type of vascular disease or surgical indication (aortic, peripheral arterial, or retroperitoneal tumor), will be recorded prior to group assignment to facilitate stratified randomization. Within several days prior to the intervention, baseline laboratory parameters will be assessed, including hemoglobin, albumin, cardiac biomarkers, serum creatinine (SCr), and estimated glomerular filtration rate (eGFR). Baseline serum creatinine is defined as the most recent stable serum creatinine measurement obtained within 30 days before surgery, typically from the admission assessment or the preoperative evaluation performed 24 to 48 h before surgery. For patients with multiple preoperative measurements, the most recent lowest stable value before skin incision will be used as baseline. For the rare patients without a preoperative baseline value, the baseline creatinine will be estimated by back-calculation using the MDRD equation, assuming a baseline eGFR of 75 mL/min/1.73 m^2^, as recommended by the KDIGO guidelines. Postoperative serum creatinine will be measured systematically at 48 h (±4 h) and 72 h (±4 h) after surgery, as well as on postoperative day 7 or at discharge, whichever occurs first. Additional measurements will be performed whenever clinically indicated. During indwelling urinary catheterization, including the intraoperative period and ICU stay, hourly urine output will be recorded continuously to evaluate the KDIGO urine output criterion. After transfer to the general ward and removal of the urinary catheter, the primary determination of AKI will rely strictly on the serum creatinine criterion, because self-voided urine output recording is inherently imprecise in routine clinical practice. To maximize reproducibility, the primary analysis will use the creatinine-only KDIGO criteria, while the combined criteria incorporating urine output during indwelling catheterization will be reported as a supportive sensitivity analysis. Anatomic characteristics will be derived from measurements obtained via CTA. The following perioperative parameters will be recorded: operative duration, surgical approach, estimated intraoperative blood loss, red blood cell transfusion volume, and postoperative serological test results.

A standardized Case Report Forms (CRFs) will be employed by trained investigators for data collection. Patient demographics, comorbidities, imaging-based anatomical parameters, and laboratory results will be extracted from the hospital's electronic patient registry and Picture Archiving and Communication System (PACS). To ensure data accuracy, all extracted data will be independently cross-verified by two investigators. Follow-up data will be actively collected via telephone and the WeChat platform to maximize participant retention and follow-up completeness. Detailed data management procedures are available from the monitoring committee upon request.

### Statistical analysis

All statistical analyses will be performed using R software (version 4.5.1). Categorical variables will be summarized as frequencies (percentages). Continuous variables will be presented as mean ± standard deviation (SD) for normally distributed data, or as median (interquartile range, IQR) for non-normally distributed data. Baseline characteristics and 30-day perioperative outcomes will be compared between the two groups. Variables including sex, age, smoking status, functional status, and preoperative comorbidities (e.g., hypertension, diabetes, coronary artery disease, cancer, and chronic lung disease) will be considered potential confounders.

This trial is designed as a prospective, randomized, controlled superiority study to evaluate whether DRIPC significantly reduces the 30-day incidence of MACCE compared with standard care alone. The primary hypothesis is that the 30-day incidence of the composite primary endpoint will be significantly lower in the DRIPC group than in the control group. All primary and secondary outcome analyses will be conducted under a superiority framework using two-sided hypothesis tests at a significance level of *α* = 0.05. The primary binary endpoint is the treatment effect of DRIPC on the occurrence of composite MACCE within 30 days postoperatively in all randomized participants regardless of adherence (treatment policy strategy), quantified as the odds ratio (OR) and absolute risk difference. Accordingly, the confirmatory primary efficacy analysis will be conducted strictly in the ITT/mITT population, including all randomized participants according to their assigned treatment group. To account for baseline risk heterogeneity across distinct anatomical vascular territories (including aortic, peripheral arterial, carotid, visceral, and retroperitoneal tumor-related procedures), disease and surgical categories will be incorporated into the primary statistical model as the fundamental stratification factor. The primary endpoint (30-day MACCE incidence) and key binary secondary endpoints (e.g., incidence of acute kidney injury) will be evaluated using stratified Cochran-Mantel-Haenszel (CMH) tests and multivariable stratified logistic regression models with disease and surgical categories specified as the stratification factor.

In the primary analysis, MACCE is evaluated as a composite binary outcome. To explicitly address the differing clinical severity among individual components without relying on arbitrary weighting, a hierarchical composite endpoint analysis using the Finkelstein-Schoenfeld/Pocock win ratio approach will be conducted as a prioritized confirmatory secondary analysis for 30-day outcomes. In this pairwise comparison, 30-day events are prioritized hierarchically in order of clinical gravity: all-cause mortality, stroke, non-fatal myocardial infarction, systemic embolism, and heart failure hospitalization. The win ratio (number of winner pairs divided by loser pairs) with its 95% confidence interval (CI) and corresponding *P* value will be reported. Furthermore, all individual component event rates will be systematically recorded, tabulated, and analyzed separately to ensure complete transparency. In addition, as a prespecified secondary analysis rather than a *post hoc* analysis, multivariable logistic regression models will be performed to adjust for predefined baseline prognostic covariates, including age, sex, baseline renal function (eGFR), and key cardiovascular comorbidities, thereby rigorously evaluating the independent treatment effect of DRIPC on 30-day MACCE risk.

To test the robustness of the primary findings and explore treatment heterogeneity, the following sensitivity and exploratory analyses will be conducted. First, both mITT and PP analyses will be evaluated. The mITT analysis serves as the primary confirmatory analysis, whereas the PP analysis will be performed as a sensitivity analysis excluding patients with major protocol violations, non-compliance, or crossover. For secondary outcomes assessed at predefined follow-up time points, normally distributed continuous variables will be compared using the independent samples t-test, non-normally distributed continuous variables using the Mann–Whitney U test, and categorical variables using the chi-square test or Fisher's exact test. Effect estimates (*β* coefficients or odds ratios [ORs]) with 95% CIs will be derived using generalized linear models (GLMs). Second, propensity score matching (PSM) will not be used in the primary analysis, but is prespecified solely as an ancillary sensitivity analysis (1:1 nearest-neighbor matching without replacement, calipe*r* = 0.2 SD of logit PS) in the following scenarios: (i) in the presence of chance baseline imbalances (standardized mean difference >0.10) in key prognostic covariates despite stratified randomization; (ii) in the PP population; and (iii) in evaluating potential confounding arising from non-random loss to follow-up. Missing data will be handled using multiple imputation techniques under the assumption of missing at random. Third, subgroup analyses for 30-day MACCE will be performed according to prespecified variables: surgical cohort (aortic, peripheral arterial, or retroperitoneal tumor); degree of coronary or carotid artery stenosis (<50% vs. ≥ 50%); sex (male vs. female); age (≤70 years vs. > 70 years); and surgical approach (endovascular vs. open surgery). Within each subgroup, odds ratios (ORs) and 95% CIs will be estimated using multivariable logistic regression models. Interactions between treatment and subgroup will be formally assessed using likelihood ratio tests, and the corresponding *P* values for interaction will be reported to determine whether the protective efficacy of DRIPC differs significantly across strata. The results will be summarized in a forest plot.

A dedicated Data and Safety Monitoring Board (DSMB) has been established, comprising independent biostatisticians, vascular surgeons, clinical investigators, and research coordinators. Substantial protocol amendments, such as adjustments to sample size or eligibility criteria, will require formal notification and approval by the DSMB. In contrast, non-substantial modifications will be documented by the research team without immediate DSMB review.

### Monitoring and protocol amendments

The DSMB and the Institutional Review Board (IRB) retain the authority to audit all collected data periodically and may raise concerns regarding the study's scientific validity or ethical conduct at any time. An independent statistician, not otherwise involved in the trial, will perform the prescribed interim analysis according to a pre-specified charter.

The study may be subject to modification, suspension, or termination based on the recommendations of the IRB or DSMB. The trial will be terminated prematurely if any of the following conditions are met: (a) outcomes in the control group are demonstrated to be significantly superior to those in the intervention group; (b) occurrence of serious adverse events definitively attributed to the study intervention; or (c) emergence of other unforeseen effects resulting from the trial intervention or procedures that raise significant ethical or safety concerns.

## Declarations

All research team members will sign a confidentiality agreement with the principal investigator. Furthermore, all team members will treat study-related reports, meeting discussions, and minutes as confidential. Participants’ identifying information will be encrypted to prevent data leakage throughout the study period. The principal investigator will be required to maintain adequate records to ensure that the conduct of the trial is fully documented. All essential documents related to the conduct of this trial must be retained by the principal investigator for a minimum period of 15 years.

## Patient and public involvement

Patient engagement has been incorporated into the trial design. Patient enrollment has not yet commenced. When enrollment commences, prospective participants will be clearly informed that the study protocol has undergone formal peer review. A pre-trial survey administered to previous patients directly informed the selection of key patient-centered outcomes, including health-related quality of life and time to return to normal activities, as well as the feasibility of postoperative follow-up via WeChat and telephone.

## Discussion

The overall burden of vascular disease increases progressively with advancing age. Patients undergoing major vascular and retroperitoneal tumor-related vascular surgeries are exposed to a substantial burden of perioperative morbidity and mortality. Intraoperative hemodynamic instability, visceral and limb ischemia-reperfusion injury, and iodinated contrast-induced nephrotoxicity collectively predispose this comorbid cohort to MACCE and AKI. Vascular procedures remain designated as high-risk interventions in consecutive ACC/AHA guidelines, while contemporary ESC guidelines underscore that concomitant peripheral arterial disease confers a 4- to 5-fold surge in perioperative MACCE risk (1, 2, 17). Furthermore, Global Burden of Disease estimates reveal an 82.1% increase in aortic aneurysm-related mortality over the past three decades, highlighting a persistent global public health challenge (17). Consequently, patients undergoing vascular surgery face substantially elevated perioperative risks of myocardial infarction, AKI, and stroke, thereby underscoring the critical importance of optimized perioperative management and effective organ protection strategies.

Although RIPC has demonstrated organ-protective efficacy in selected surgical cohorts, clinical trial results in vascular surgery remain inconsistent. Conventional acute RIPC protocols, which are typically administered immediately before or during surgery, may have limited efficacy because of pharmacological confounding, particularly the potential attenuation of ischemic conditioning signaling by propofol anesthesia (18–22).

To overcome these limitations, DRIPC has emerged as a promising strategy. Administered 24 to 72 h preoperatively, DRIPC activates the “second window of protection” (SWOP), a robust cytoprotective state driven by *de novo* gene transcription and the upregulation of inducible protective mediators such as inducible nitric oxide synthase (iNOS), cyclooxygenase-2 (COX-2), and heat shock proteins. This preoperative window provides sustained multi-organ protection that spans the critical perioperative period while avoiding the confounding effects of intraoperative anesthetics (9, 10), but is also associated with inconsistent clinical evidence. Nonetheless, translational evidence for DRIPC remains predominantly confined to cardiac surgical cohorts with mixed findings. For instance, large randomized trials by Kim et al. and Jia et al. in cardiac surgery failed to demonstrate significant reductions in postoperative myocardial infarction or composite clinical endpoints (23, 24). In contrast, a study involving patients presenting with acute myocardial infarction demonstrated that DRIPC improved the myocardial salvage index in those experiencing their first infarction (25). The negative findings in cardiac surgery may be heavily confounded by cardiopulmonary bypass-induced systemic inflammatory storms, which might mask or overwhelm the subtle protective signal of DRIPC. Furthermore, preliminary preclinical and clinical evidence suggests that remote conditioning may also protect against contrast-induced nephropathy (25–27) Collectively, these evidences suggest that DRIPC holds considerable promise for patients undergoing major vascular surgery and warrants further rigorous prospective investigation.

To our knowledge, this study is one of the earlist prospective, randomized controlled superiority trials specifically designed to evaluate the clinical efficacy and safety of DRIPC in patients undergoing major vascular surgery. The principal methodological strengths of this protocol include: (1) an intention-to-treat framework with disease- and procedure-stratified randomization across distinct vascular territories (aortic, peripheral arterial, carotid, and retroperitoneal vascular procedures); (2) a prespecified hierarchical composite outcome analysis using the Finkelstein-Schoenfeld/Pocock win ratio approach to account for the differential clinical severity of MACCE components; and (3) a rigorous 30-day binary incidence and safety evaluation. Nevertheless, this trial has several limitations that warrant acknowledgment. First, as a single-center trial, variations in surgical volume and perioperative care protocols may limit the generalizability of the findings to other institutions. Second, urine output quantification following catheter removal is inherently imprecise, which may compromise the accuracy of AKI diagnosis. Finally, given the broad spectrum of diseases encompassed by this trial, standardized postoperative management strategies could not be uniformly applied to all participants. Consequently, the potential bias introduced by this clinical heterogeneity, as well as its impact on the study outcomes, remains undetermined.

In conclusion, this trial is designed to systematically evaluate the impact of DRIPC on the incidence of perioperative major adverse cardiac and cerebrovascular events, acute kidney injury, all-cause mortality, and other critical clinical outcomes in patients undergoing major vascular surgery. The findings of this trial are expected to provide Level I evidence concerning the efficacy and safety of this non-invasive intervention in the context of major vascular surgery.

## Patient and public involvement

Patient enrollment has not yet begun. Upon initiation of enrollment, it will be clearly communicated that the study protocol has undergone peer review. Details regarding their involvement are provided in the Methods section.
